# Effects of defined organic layers on the fluorescence lifetime of plastic materials

**DOI:** 10.1007/s00216-025-05888-y

**Published:** 2025-05-09

**Authors:** Nina Leiter, Maximilian Wohlschläger, Martin Versen, Sonja D. Harter, Tina Kießlich, Franziska Lederer, Stefanie Clauß, Dietmar Schlosser, Emanuel Gheorghita Armanu, Christian Eberlein, Hermann J. Heipieper, Martin G. J. Löder, Christian Laforsch

**Affiliations:** 1 Faculty of Engineering Sciences, Rosenheim Technical University of Applied Sciences, Hochschulstraße 1, 83024 Rosenheim, Germany; 2 Biotechnology Department, Helmholtz Institute Freiberg for Resource Technology, Helmholtz-Zentrum Dresden-Rossendorf, Bautzner Landstraße 400, Dresden, 01328 Germany; 3 Department of Applied Microbial Ecology, Helmholtz Centre for Environmental Research – UFZ, Permoserstraße 15, Leipzig, 04318 Germany; 4 Department of Molecular Environmental Biotechnology, Helmholtz Centre for Environmental Research – UFZ, Permoserstraße 15, Leipzig, 04318 Germany; 5 Animal Ecology I and BayCEER, University Bayreuth, Universitätsstraße 30, Bayreuth, 95440 Germany

**Keywords:** Plastic identification, Microplastic, Organic contamination, Fluorescence lifetime, FD-FLIM

## Abstract

Plastics have become an integral part of modern life, and linked to that fact, the demand for and global production of plastics are still increasing. However, the environmental pollution caused by plastics has reached unprecedented levels. The accumulation of small plastic fragments—microplastics and nanoplastics—potentially threatens organisms, ecosystems, and human health. Researchers commonly employ non-destructive analytical methods to assess the presence and characteristics of microplastic particles in environmental samples. However, these techniques require extensive sample preparation, which represents a significant limitation and hinders a direct on-site analysis. In this context, previous investigations showed the potential of fluorescence lifetime imaging microscopy (FLIM) for fast and reliable identification of microplastics in an environmental matrix. However, since microplastics receive an environmental coating after entering nature, a challenge arises from organic contamination on the surface of microplastic particles. How this influences the fluorescence signal and the possibility of microplastic detection are unknown. To address this research gap, we exposed acrylonitrile butadiene styrene (ABS) and polyethylene terephthalate (PET) plastic samples to peptides, proteins, bacteria, and a filamentous fungus to induce organic contamination and mimic environmental conditions. We analyzed the fluorescence spectra and lifetimes of the samples using fluorescence spectroscopy and frequency-domain fluorescence lifetime imaging microscopy (FD-FLIM), respectively. Our results demonstrate that reliably identifying and differentiating ABS and PET was possible via FD-FLIM, even in the presence of these biological contaminations. These findings highlight the potential of this technique as a valuable tool for environmental monitoring and plastic characterization, offering a rapid and efficient alternative to currently used analytical methods.

**Graphical Abstract:**

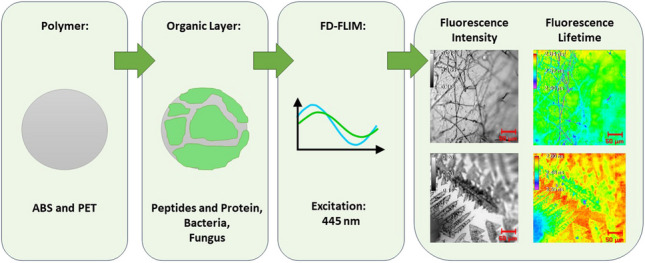

**Supplementary Information:**

The online version contains supplementary material available at 10.1007/s00216-025-05888-y.

## Introduction

Plastics are an integral part of modern life due to the great advantages this family of materials provides and recently reached global production rates of more than 400 million annual tons [1]. The importance of plastics as single-use packaging material leads to the fact that plastic waste often ends up in our environment, and in this context especially the accumulation of micro- and nanoplastics (MPs and NPs) particles gained great attention in science and public. Meanwhile, it is recognized that this new group of contaminants potentially poses a severe threat to organisms, our ecosystems, and also human health [2–8]. Hereby, MP polymer type, shape, size, and furthermore, surface characteristics potentially determine the transport and accumulation space in the respective environmental compartment, the interaction with organisms, and the toxicity of MPs. Consequently, as a data basis for an ecological risk assessment, information on at least the number, plastic type, shape, and size during the analysis of environmental MP samples is necessary [9].

Predominant mass-based analytical methods are thermal extraction and desorption-gas chromatography/mass spectroscopy (TED-GC/MS) [10, 11] or pyrolysis GC/MS [12, 13]. These methods are used to determine the mass proportion of different plastic species in a collected environmental sample. The disadvantage is that the required parameters for the number, size, and shape of the MP, which are essential for risk assessment, cannot be determined. In addition, both methods are destructive, meaning that the sample cannot be analyzed further.

The most commonly used non-destructive analytical methods to assess the necessary MP parameters are micro-Fourier transform infrared (FTIR), attenuated total reflectance (ATR) FTIR, and Raman spectroscopy. In order to analyze MP samples using these methods, several time-consuming extraction and purification steps must be carried out [6]. Consequently, the application of these spectroscopic methods for detecting MPs directly in environmental samples is hindered, impeding high-throughput analyses and rapid evaluations of contamination levels [14].

Therefore, there is an urgent need to develop innovative solutions or novel methods that can improve the efficiency of plastics analysis and direct in situ application. Such advancements would not only facilitate the rapid and high-throughput examination of a more significant number of samples but also mitigate the current limitations faced by existing techniques. Implementing in situ analysis approaches, in particular, holds promise for overcoming the current challenges of MP analytics.

Building on the challenges of existing analytical methods, researchers have explored the potential of fluorescent markers as an innovative approach to improve plastic identification in recycling processes and MP analysis [15, 16]. However, the sample preparation is time consuming, and it is also crucial to note that the long-term health implications of these fluorescence markers have yet to be thoroughly investigated, necessitating further research to ensure their safety and efficacy. Studies have shown that even conventional plastics emit fluorescence when excited by near-UV light [17, 18]. This phenomenon has been observed in polymers such as polypropylene (PP) and polyethylene (PE) despite their lack of low-lying chromophores. The unexpected fluorescence is attributed to the presence of chain breaks and oxidative byproducts resulting from the manufacturing process of these technical polymers [19–21]. Consequently, MP in the environment is expected to exhibit characteristic fluorescence responses, potentially offering a novel method for their detection and identification in complex environmental matrices.

According to previous works, the determination of the fluorescence lifetime is possible using both time-domain (TD) and frequency-domain (FD) fluorimetry [22]. TD fluorimetry has shown promise in determining the size and shape of various plastics [23] as well as analyzing plastics in aquatic environment matrixes [21]. Initial investigations using FD fluorimetry demonstrated the feasibility of identifying and differentiating plastics from environmental materials [24]. Further advancements have been made in classifying plastics using neural networks trained on FD fluorescence lifetime imaging microscopy (FD-FLIM) data [25] and automating the FD-FLIM measurement procedure [26].

FD fluorimetry offers advantages in terms of efficiency, featuring shorter integration times while integrating over the entire fluorescence spectrum. This capability enables rapid data acquisition and analysis, making FD fluorimetry particularly suitable for high-throughput applications [25].

An important aspect of detecting plastics in environmental matrices is the biological contamination of samples. The effects of organic layers on FD fluorimetry represent a significant research gap that warrants closer examination. To address this important research question, the present study aimed to investigate the effects of organic layers’ presence on the accuracy and reliability of FD fluorimetry in identifying plastics, thereby contributing to a more robust understanding of the challenges posed by biological contaminants in environmental monitoring efforts.

To assess the influence of organic layers on plastic analysis using FD-FLIM, plastic samples of acrylonitrile butadiene styrene (ABS) and polyethylene terephthalate (PET) were coated with peptides, proteins, bacteria and a filamentous fungus under controlled conditions and measured via FD-FLIM after different times of incubation. While peptide monolayers form up to 2 nm layers at a sample surface, the protein bovine serum albumin (BSA) forms a monolayer of about 14 nm [27]. Depending on the time, thicker layers of bacteria or fungus form stable biofilms at surfaces such as polymers. Analyzing the fluorescence spectrum and FD-FLIM data provided insights into whether plastics can still be detected despite the presence of the defined organic layers, improving our understanding of the interaction between different biological contaminants and fluorimetry techniques.

## Theory

A description of the theory behind FD-FLIM was discussed in detail by Wohlschläger et al. [25]. The fluorescence lifetime is defined as the duration required for the intensity of a material’s fluorescence signal to diminish to 1/e of its initial value induced by the short and highly energetic excitation pulse and the exponentially decreasing fluorescence lifetime [22, 25]. In the FD, the light source is sinusoidally or rectangularly modulated, having a defined modulation frequency* ω*. The fluorescence signal follows the sinusoidal/rectangular excitation with a phase shift *φ*. The fluorescence signal is also attenuated in its amplitude and shifted in its average value and is described by the amplitude of excitation *B*, the amplitude of the fluorescence emission *b*, the average value of excitation *A*, the average value of the fluorescence emission *a*, and the phase shift *φ*. Using the defined modulation frequency ω and the phase shift *φ*, the phase-dependent fluorescence decay time ${\tau }_{\text{PH}}$ can be calculated according to Eq. (1). By measuring the values* A*,* a*,* B*, and *b*, the modulation index *M* can be calculated (Eq. (2)), and therefore the modulation-dependent fluorescence lifetime ${\tau }_{\text{M}}$ (Eq. (3)) [22, 25].

1 $${\tau }_{PH}=\frac{\text{tan}\left(\varphi \right)}{\omega }$$

2 $$M=\frac{(b/a)}{(B/A)}$$

3 $${\tau }_{M}=\frac{\sqrt{\frac{1}{{M}^{2}}-1}}{\omega }$$

With an FD-FLIM camera, the location-dependent *φ* and *M* and the corresponding fluorescence lifetimes can be measured. ${\tau }_{PH}$ and ${\tau }_{M}$ can be calculated. Thus, the result of an FD-FLIM measurement is an image stack of 5 images: fluorescence intensity *I*, phase shift *φ*, modulation index *M*, phase-dependent fluorescence lifetime ${\tau }_{PH}$ and modulation-dependent fluorescence lifetime ${\tau }_{M}$ [25]. Depending on the material, multiple fluorescence signals may overlap during fluorescence measurement, resulting in the appearance of multiple maxima in a Gaussian analysis of the fluorescence lifetime distribution.

## Materials and methods

### Incubation methods

For this study, two different types of plastic granulates were selected as base materials: acrylonitrile butadiene styrene (ABS; Novodur P2MC) and polyethylene terephthalate (PET; “Type M” Trevira). The individual granulates have a diameter of approximately 3 mm. ABS and PET are among the most frequently used base materials for application-related plastic components and have already been analyzed in previous studies by Wohlschläger et al. [25, 28]. The thermoplastic ABS is applied in majority within the electronic and automotive industries [29]. Resulting from improper disposal of related waste, it has been reported as a major microplastic contaminant in soils of e-waste dismantling sites, and is also known from freshwater as well as marine environments [30, 31]. Within the BMBF project PepMetal, ABS-binding peptides were identified for the substitution of chromium (VI) in the galvanization sector. One of these peptides was applied in the current study to observe the impact of a very thin biomolecule layer on the FLIM polymer detection. As one of the four most produced polymers in the world, PET is used in majority in textile and bottle applications and is highly abundant in environmental samples. PET is biodegradable [32], but nevertheless PET micro- and nanoplastics are known as prominent contaminants of water bodies, sediments, dust and biota [33]. Due to these reasons, PET was chosen in this study as target polymer for the biofilm formation using proteins, bacteria and fungus. The granulates were incubated with peptides, bacteria, and filamentous fungus. The maximum incubation times were adjusted according to the different behaviors of peptides, bacteria, and filamentous fungus.

#### Peptides and protein

Forty pellets of the plastic type ABS were incubated with a putative ABS-binding 12-mer peptide (molecular weight 2 Dalton), previously identified via phage surface display technology (Ph.D-12 Phage Display Library, E8110S, New England Biolabs, Inc., Ipswich, MA, USA) in a total volume of 7.5 ml and a peptide concentration of 2 mg/ml. Depending on the incubation time, the formation of a 2 nm peptide monolayer at the surface of ABS pellet surfaces was expected without further influences on degradation, coloring or autofluorescence. Further, forty pellets of the plastic type PET were incubated in 7.5 ml of a 10 mg/ml solution of the 66.4 kDa protein Bovine Serum Albumin (BSA, 1.12018, Sigma Aldrich, Merck KGaA, Darmstadt, Germany). BSA is known for its good adsorption characteristics on hydrophilic as well as hydrophobic surfaces [34] and is expected to form a 14 nm monolayer at the surface of the PET particles without any further influences such as degradation, coloring or autofluorescence. Incubation was carried out in an overhead shaker at room temperature (RT). The sampling for fluorescence analysis was performed at seven defined times: t1 = 5 min, t2 = 10 min, t3 = 20 min, t4 = 2 h, t5 = 4 h, t6 = 16 h and t7 = 48 h. Four pellets were removed from the suspension each, transferred into a 96-well plate, and surplus liquid evaporated overnight at RT. Additionally, four granulates representing the time t0 were not incubated and used as a reference not covered with peptides and protein.

#### Bacteria

In addition, the incubation of ABS and PET with a bacterial culture mainly consisting of strains of *Pseudomonas canadensis* and *Microbacterium ginsengisoli* was selected because it was isolated within the research of the FINEST project for its capability to efficiently degrade phthalic acid ester plasticizers. Apart from adsorption on hydrophobic surfaces such as ABS and PET, no other interactions with the surfaces studied were expected. Bacteria were cultivated in a modified Hartmans mineral medium as previously described [35] with 4 g/L Na_2_ succinate as sole carbon and energy source in 50 ml shake flasks. Experiments were started from an overnight culture with a titer of 1*10^8^ cells/ml (optical density OD at 560 nm: 0.1), and cultures were incubated in the presence of 30 granulates of PET or ABS granules. From these cultures, four granulates were taken for fluorescence analysis at t0 = 0 h, t1 = 2 h, t2 = 4 h, t3 = 8 h, t4 = 16 h, t5 = 32 h. After 32 h, the bacterial biofilm already fully covers the plastic granules. For scanning electron microscopy (SEM) analysis, four additional granulates were taken after 0 and 32 h of incubation.

#### Fungus

The incubation of ABS and PET with the filamentous basidiomycete *Trametes versicolor* DSM 11269, representing an environmentally frequent fungal white-rot species known to degrade a wide range of different organic environmental pollutants but not expected to cause further biochemical alteration of ABS and PET surfaces beyond adsorption of its biomass [36–38], was conducted as follows. The fungus was pre-cultured on malt extract agar plates (1% malt extract, 1.5% agar; pH 5.7) at 30 °C for 1 week. To prepare the fungal inoculum, five agar plugs (1 cm in diameter) derived from the edge of fungal colonies were homogenized in 5 ml of a liquid 1% malt extract (w/v) medium containing 50 µM CuSO_4_, 50 µM MnSO_4_, and 20 µM FeSO_4_ in addition (pH 5.7) with the help of an Ultra-Turrax (IKA-Werke GmbH & CO. KG, Staufen, Germany). For exclusive coating with fungal mycelium, ABS and PET granules were heat-treated at 70 °C overnight in a drying oven in order to minimize potential microbial contamination. Thereafter, ABS or PET granules (5–7 per well) were aseptically placed in the wells of 24-well microplates (flat bottom, sterile, with lid; NUNCLON™, Thermo Fisher Scientific, Waltham/MA, USA), covered in 700 µl of the aforementioned liquid malt extract medium, and inoculated with 70 µl of the prepared fungal inoculum. After capping the following inoculation, plates were additionally sealed with parafilm to minimize evaporation and then incubated at 30 °C in the dark. At each time point (t0 = 0 min, t1 = 4 h, t2 = 24 h, t3 = 48 h, t4 = 72 h, t5 = 144 h, t6 = 168 h, and t7 = 240 h), four granules of ABS and four granules of PET were taken for fluorescence analysis, with an additional granule from each material for SEM analysis. In addition, controls were included for each time point, with ABS and PET granules incubated in malt extract medium, without the addition of fungal inoculum.

### Evaluation

#### Experimental setup

The experimental setup is based on a system developed by Wohlschläger et al. [24]. The apparatus is centered around an EPAF150 microscope (Cascade Microtech), providing 20 × magnification. Fluorescence excitation in the samples is achieved by wide field illumination using a laser diode PhoxX + 445–500 (Omicron-Laserage GmbH), emitting light at a wavelength of 445 nm with an optical output power of 500 mW. The detection of the fluorescence signal is accomplished through two interchangeable components: a minispectrometer (Hamamatsu Photonics GmbH) for measuring the spectral fluorescence response and a pco.flim camera (Excelitas PCO GmbH) for capturing FD-FLIM images. Filters are integrated into both the excitation and emission light paths to optimize signal quality. The excitation clean-up filter (exciter, AHF Analysetechnik) narrows the excitation light’s bandwidth to 10 nm, while the emission longpass filter (emitter, AHF Analysetechnik) blocks unwanted scattered light and reflections shorter than 460 nm. This filter configuration ensures that only the fluorescence signal is captured by the camera, thereby enhancing the precision of the measurements. The area of measurement of the pco.flim camera contains 1004 × 1008 pixels. Considering the field of view of (0.28 × 0.28) mm^2^ and the detector pixel size of the pco.flim camera of 5.6 µm, a pixel resolution of approximately 0.28 µm is given. Spectral measurements by spectrometer are averaged over the field of view.

#### Measurement procedure and evaluation of fluorescence spectra

**Fig. 1:**
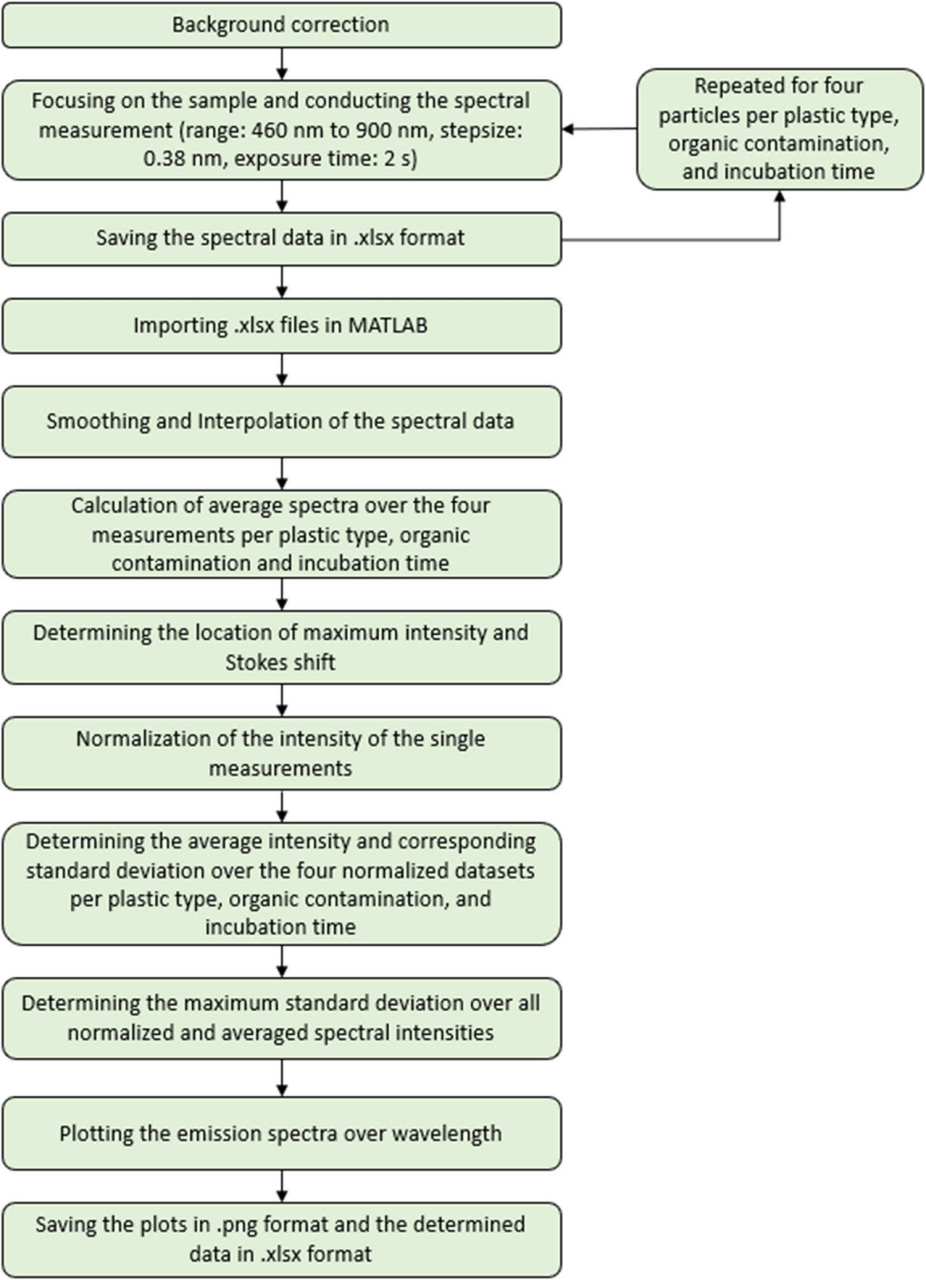
Flowchart of the measurement procedure and evaluation of fluorescence spectra

An overview of the steps of the measurement procedure and evaluation of the fluorescence spectra is presented in Fig. 1. The spectral measurement began by setting the laser power to 100% (500 mW) and the exposure time to 2 s The wavelength range for the measurements was specified from 460 to 900 nm with a stepsize of 0.38 nm. An initial background measurement against air was conducted and subsequently subtracted from the spectral measurements. The sample was focused and the spectrum measurement was recorded. The spectral data were stored in a table. This process of focusing, measurement and data storage was repeated until four measurements (i.e., four granulates) per plastic type, organic contamination, and incubation time were completed.

The data analysis of fluorescence spectra was conducted following the methodology outlined by Wohlschläger et al. [24]. Initially, the four measurements per plastic type, organic contamination, and incubation time were imported into MATLAB. The resulting spectral histograms were subsequently smoothed using a local regression model with weighted linear least squares and a second-degree polynomial model and interpolated with a step size of 0.01 nm to ensure a refined data representation. Then, the average curve of the four histograms was calculated from these four individual curves. The next step involved is the determination of the location of the maximum intensity of the averaged spectra (i.e., Counts) alongside with its corresponding wavelength (${\lambda }_{\text{max}}$). This information was crucial for estimating the Stokes shift (${\Delta \lambda }_{\text{max}}$), which is calculated by subtracting the excitation wavelength (i.e., absorbance maximum) from the wavelength of the emission maximum. The maximum intensity values and the Stokes shift were systematically recorded in a table. The normalized and averaged fluorescence spectrum distribution was determined by normalizing the intensity of the four single measurements per plastic type, organic contamination, and incubation time and then calculating the mean intensity value and standard deviation depending on the wavelength over these four measurements. In addition, the maximum value of the calculated standard deviation over all averaged spectral distributions was determined. Finally, the averaged curves were graphically represented, displaying absolute and normalized fluorescence intensity as a function of wavelength to facilitate a clear visualization of the results.

#### Measurement procedure and evaluation of FD-FLIM

**Fig. 2:**
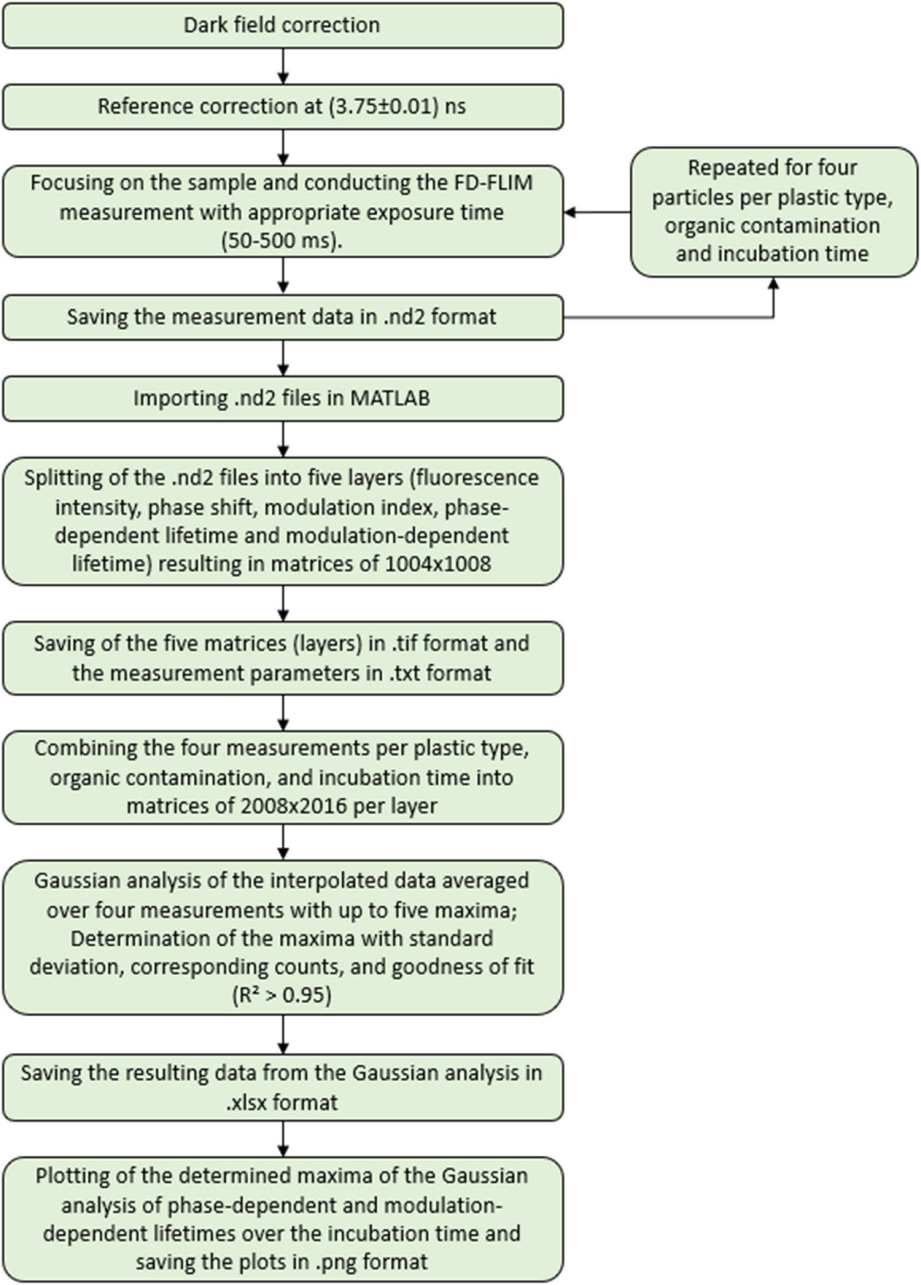
Flowchart of the measurement procedure and evaluation of FD-FLIM

An overview of the steps of the measurement procedure and evaluation of the FD-FLIM data is presented in Fig. 2. Prior to the sample measurement, the measurement parameters were chosen: the modulation frequency was set to 30 MHz, a rectangular signal was selected, and the laser power was adjusted to 100% (500 mW). Following this, the measurement system was calibrated using a reference plate (Starna Scientific) with a known fluorescence lifetime of 3.75 ns, resulting in a statistical uncertainty of ± 0.01 ns in the fluorescence lifetimes. The exposure time was then adjusted appropriately, ranging between 50 and 80 ms for ABS and between 150 and 500 ms for PET. The sample measurement was carried out, and data on fluorescence intensity, phase shift, modulation index, and both phase- and modulation-dependent lifetimes were saved in TIF files. After each measurement, the particle was changed. This sequence of adjusting the exposure time, measuring, and saving data was repeated until a total of four measurements (i.e., four granulates) per plastic type, organic contamination, and incubation time were completed.

The FD-FLIM data evaluation was conducted in accordance with Wohlschläger et al. [25]. For data analysis, the four measurements for each plastic type, organic contamination and incubation time were imported into MATLAB, encompassing the five image layers: fluorescence intensity, phase shift, modulation index, phase-dependent lifetime, and modulation-dependent lifetime. Each layer consists of 1004 × 1008 values. These measurement values were consolidated into matrices of 2008 × 2016 data points for each layer. A histogram for each layer was generated from these matrices. Subsequent Gaussian analysis of the histograms involved calculating the maxima, mean, and standard deviation for each layer over four measurements. The result of the Gaussian analysis could consist of up to five fitted Gaussian curves (i.e., five maxima, means, and standard deviations per layer). Regarding the results to be discussed in this research, this statistical evaluation led to a maximum of two fitted Gaussian curves. A goodness of fit test was performed to evaluate the accuracy of the Gaussian fits ($${R}^{2}>0.95)$$. The values from the Gaussian analysis were systematically recorded in a table. Additionally, the phase- and modulation-dependent lifetime mean values $${\tau }_{\text{PH}1}$$, $${\tau }_{\text{PH}2} ,{\tau }_{\text{M}1}$$, $${\tau }_{\text{M}2}$$ and corresponding standard deviations (i.e.,$$1*\sigma$$) from the Gaussian analysis over the incubation time were illustrated in a logarithmic format.

#### Scanning electron microscopy (SEM) analysis

In order to acquire high-quality images of the immobilized bacterial consortium (*Pseudomonas canadensis* and *Microbacterium ginsengisoli*) and the filamentous fungus *T. versicolor* on the MPs (ABS and PET) beds surface, a field-emission SEM (Merlin VP Compact, Carl Zeiss, Oberkochen, Germany) with a backscattered electron image (BSE) mode coupled to an EDX spectrometer (Bruker Quantax XFlash 5060 F, Bruker Nano GmbH, Berlin, Germany), respectively, at an electron acceleration voltage of 2.0 kV and a beam current of about 248 pA was used. Thus, the immobilized surface was scanned using the in-lens electron detector negatively biased at 933 V to allow for the detection of high-energy backscattered electrons and completely suppress secondary electrons, a technique that enhances the structural features of samples and facilitates analysis processes. Each immobilized bed granule was dried and coated with a layer of 10 nm gold before SEM analysis.

## Results

The datasets of sample spectral measurements, FD-FLIM, and SEM analysis generated and analyzed during the current study are available from the corresponding author on reasonable request.

### Fluorescence spectroscopy

**Fig. 3:**
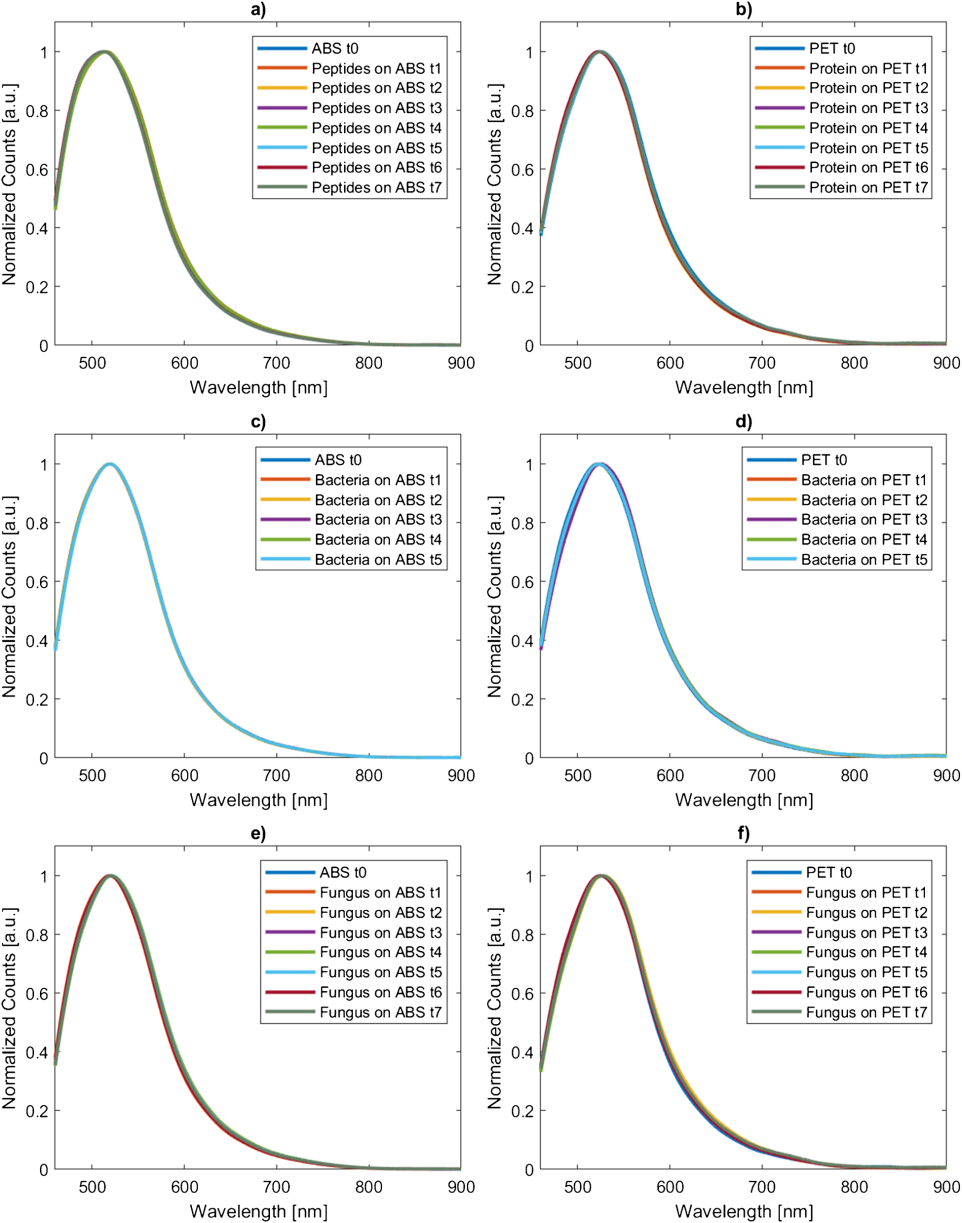
**a** Normalized and averaged fluorescence spectra of ABS extracted from peptide incubation at defined times t0–t7; **b** normalized and averaged fluorescence spectra of PET extracted from protein incubation at defined times t0–t7; **c** normalized and averaged fluorescence spectra of ABS extracted from bacteria incubation at defined times t0–t5; **d** normalized and averaged fluorescence spectra of PET extracted from bacteria incubation at defined times t0–t5. **e** normalized and averaged fluorescence spectra of ABS extracted from incubation with the fungus at defined times t0–t7; **f** normalized and averaged fluorescence spectra of PET extracted from incubation with the fungus at defined times t0–t7

The normalized and averaged fluorescence spectra of the analyzed plastic particles are shown in Fig. 3. The maximum value of the standard deviation of the normalized fluorescence spectral intensity over four measurements equals to 0.04 in arbitrary units of the Normalized Counts. Additionally, the corresponding values of the location of the maximum emission intensity (Counts, λ_max_) and Stokes shift (Δλ_max_) are listed in the Electronic Supplementary Material Table S1. The averaged fluorescence without normalization on the fluorescence intensity is shown in Electronic Supplementary Material, Fig. S2. Overall, the spectral distribution as well as the Stokes shift show no significant variation with respect to the incubation time of peptides, proteins, bacteria or fungus.

### FD-FLIM

**Fig. 4:**
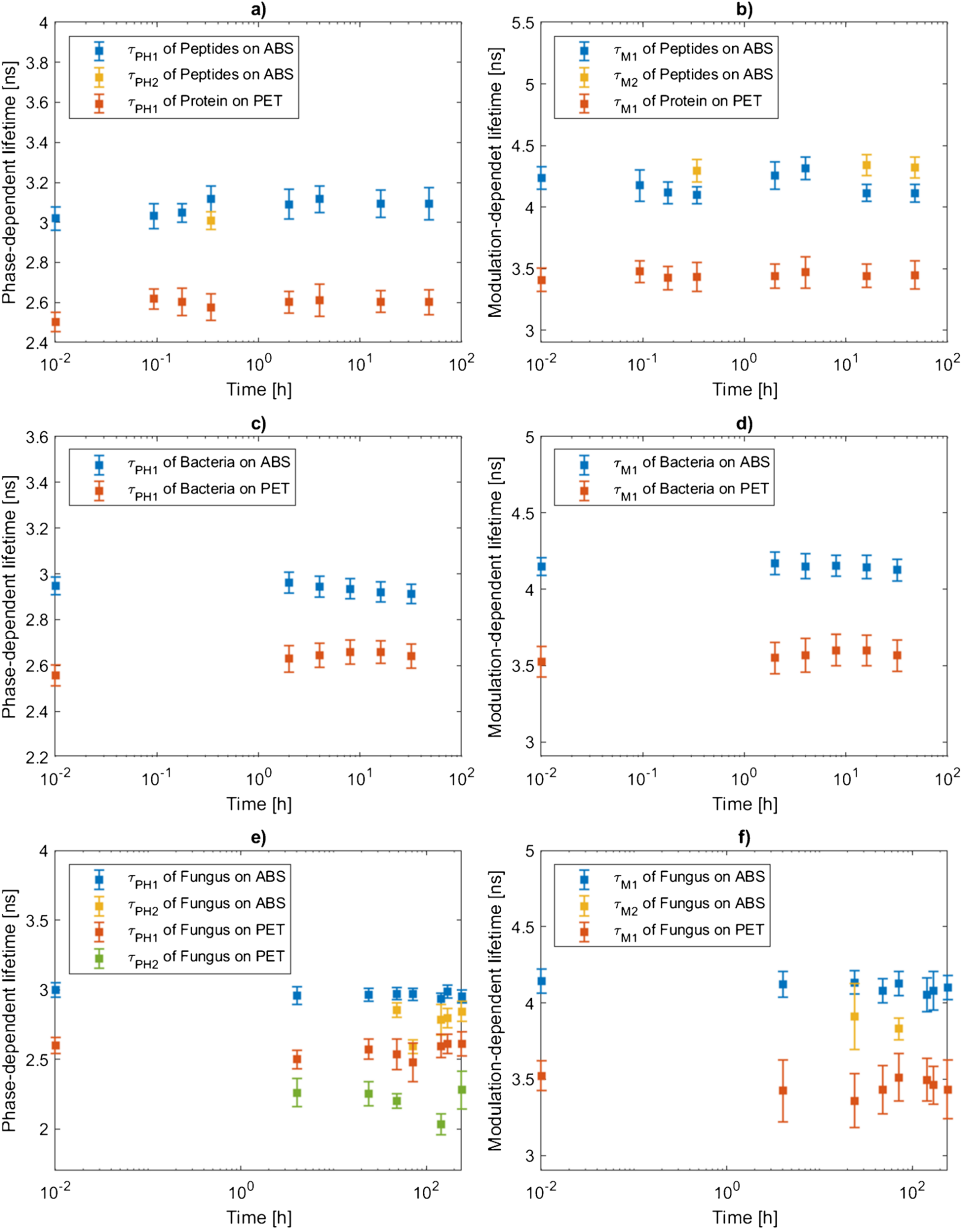
Maxima of the phase-dependent fluorescence lifetime ${\tau }_{\text{PH}1}, {\tau }_{\text{PH}2}$ determined through Gaussian analysis of ABS and PET extracted from **a** peptides or protein, **c** bacteria, and **e** fungus are shown in relation to the incubation time on a logarithmic scale. Maxima of the modulation-dependent fluorescence lifetime ${\tau }_{\text{M}1}, {\tau }_{\text{M}2}$ determined through Gaussian analysis of ABS and PET extracted from **b** peptides or protein, **d** bacteria, and **f** fungus are shown in relation to the incubation time on a logarithmic scale. The corresponding standard deviation of the maxima of the fluorescence lifetimes ($1*\sigma )$ is displayed as error bars

The study investigated the phase-dependent and modulation-dependent fluorescence lifetimes of ABS and PET substrates incubated with peptides, bacteria, and fungal mycelium to determine whether ABS and PET can be identified through fluorescence lifetimes when coated with organic layers and to understand how the fluorescence lifetimes change in the presence of organic layers. The expected maxima values of the phase- and modulation-dependent lifetimes over the time periods determined by FD-FLIM measurement and Gaussian analysis are shown in Fig. 4. Additionally, the values of the fluorescence lifetimes ${\tau }_{PH}$ and ${\tau }_{M}$ can be seen in the Electronic Supplementary Material, Table S3. Screenshots of exemplary FD-FLIM measurements of peptides, protein, bacteria and fungus on ABS and PET can be seen in the Electronic Supplementary Material Fig. S4–S9. The FD-FLIM images provide clear evidence of colonization on both ABS and PET plastic samples. From these observations, it can be concluded that peptides, fungal mycelium, and bacteria were present on the plastic samples.

#### Peptides and protein

Figure 4a shows the phase-dependent and Fig. 4b the modulation-dependent fluorescence lifetimes at different incubation times with the 12-mer peptides or BSA. The results indicate that the phase-dependent fluorescence lifetimes of peptide-coated ABS did not exhibit a significant increase. At t3, two distinct maxima were observed. Prolonged incubation times resulted in a slight rise in the phase-dependent lifetimes. The second maximum observed at t3 as well as the increased lifetimes at t4–t7, indicate a convolution of the phase-dependent fluorescence lifetimes of the peptides and ABS. For modulation-dependent fluorescence lifetimes, peptide-coated ABS displayed two maxima in three out of seven cases (see Fig. 4b). These maxima likely correspond to the lifetimes of the peptides and pure ABS. However, when considering the lifetimes at t0, no definitive conclusion can be drawn about the specific contributions of the peptides and ABS to the observed modulation-dependent lifetimes. Furthermore, PET substrates showed single maxima and slightly higher phase-dependent fluorescence lifetimes when incubated with BSA at t1–t7. However, the increase of the phase-dependent fluorescence lifetimes of approximately 0.1 ns was consistent with the results of ABS, indicating a prolongation of the fluorescence lifetime due to peptides and BSA. In contrast, it was observed that the modulation-dependent fluorescence lifetime of incubated PET remained equal throughout the incubation times without a significant impact of BSA. Additionally, from Fig. 4a and b, we observed that the distinct fluorescence lifetimes of ABS and PET allow for their identification, comparing t0 and t1–t7 and differentiation regardless of incubation time. Hence, we concluded that peptides and protein covering ABS and PET do not significantly alter the phase- and modulation-dependent fluorescence lifetimes, confirming the stability and suitability of FD-FLIM to differentiate plastics, even when coated with peptides and protein.

#### Bacteria

Figure 4c shows the phase-dependent and Fig. 4d the modulation-dependent fluorescence lifetimes at different bacteria incubation times. The fluorescence lifetimes are slightly longer at t1–t5 compared to t0. However, no significant impact of bacteria on the fluorescence lifetimes of ABS was found. Similarly, PET’s phase and modulation-dependent fluorescence lifetimes coated with bacteria also slightly prolonged the fluorescence lifetimes at t1–t5. Nevertheless, no significant changes over the incubation period or even an assignment of the fluorescence lifetime to PET and bacteria was possible.

#### Fungal mycelium

The study also explored the fluorescence lifetimes of ABS and PET plastics covered with the mycelium of *T. versicolor*. Figure 4e shows the phase- and Fig. 4f the modulation-dependent fluorescence lifetimes at different incubation times with the fungus. Gaussian analysis revealed that the phase-dependent fluorescence lifetimes of fungi-coated ABS exhibited two maxima in five out of six cases. Similarly, two maxima were identified for the modulation-dependent fluorescence lifetimes of fungus-coated ABS in two out of eight cases. These maxima of t1–t7 could be divided into two groups, corresponding to more extended phase- and modulation-dependent fluorescence lifetimes, which can be assigned to ABS when compared to t0, and shorter, which could be a convolution of the fluorescence lifetime of ABS and the fungus.

For fungus-coated PET, two maxima for the phase-dependent fluorescence lifetimes were identified by Gaussian analysis in five out of seven cases. These maxima also indicate two distinct ranges in the phase-dependent lifetimes, similar to the results seen with fungus-coated ABS and therefore underlining the founding of shorter phase-dependent fluorescence lifetimes of the fungus compared to PET. Regarding the modulation-dependent fluorescence lifetimes of fungus-coated PET, single maxima were observed, with no significant changes in decay times detected throughout the incubation period. The fluorescence lifetime data further indicate that ABS and PET can be identified and distinguished independently of fungal coverage. At t0, the longer fluorescence lifetimes are attributed to the plastic substrates. In contrast, the shorter fluorescence lifetimes are likely due to the fungal overgrowth and, thus, the convolution of the fluorescence lifetime of plastics and fungus.

### SEM analysis of the immobilized microorganisms

**Fig. 5:**
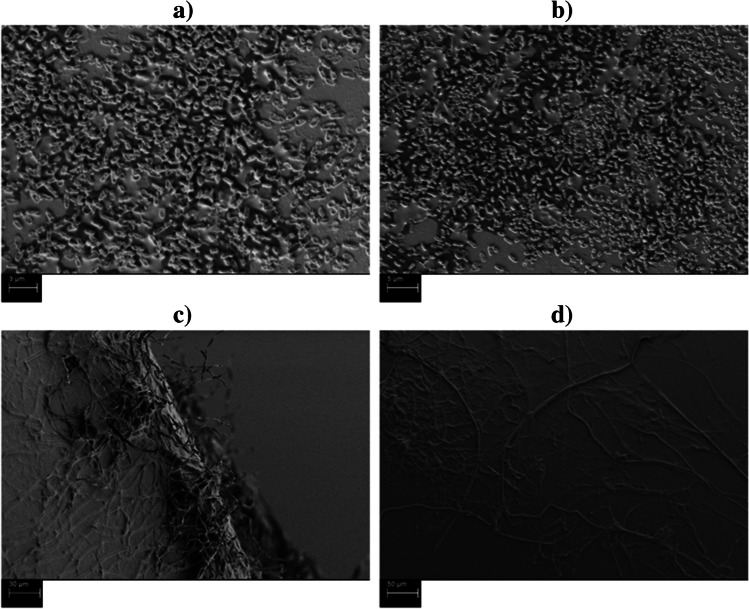
**a** SEM image of ABS extracted from incubation with the bacteria after 32 h; **b** SEM image of PET extracted from incubation with the bacteria after 32 h; **c** SEM image of ABS extracted from incubation with the fungus after 32 h; **d** SEM image of PET extracted from incubation with the fungus after 32 h

The obtained SEM images revealed smooth structures of the polymer beads (ABS and PET), with a high number of bacterial cells and—in the case of the fungus—large hyphal networks. The immobilization of the bacterial strains and the fungus is observed in Fig. 5.

It can be observed that after 32 h of incubation (30 °C) the bacterial cells are forming large biofilms on both granules, respectively on ABS (Fig. 5a) and PET (Fig. 5b), without being inhibited. Biofilm is distributed all over the granules and it covers more than 50% of the total surface area available. A filament structure (scaffold type) can be noticed for the ABS (Fig. 5c) and PET (Fig. 5 d) after 32 h of incubation (30 °C) with the filamentous fungus *T. versicolor*. Long (> 50 µm) hyphae are distributed over the polymer beds surface. The hyphal network apparently works as an anchor and penetrates the upper layers of the granules by forming stable structures.

## Discussion

Prior work has documented that several time-consuming extraction and purification steps must be carried out in order to analyze MP in environmental samples [6]. Based on studies that show that plastics emit fluorescence by near-UV excitation [17, 18], Wohlschläger demonstrated the feasibility of differentiating plastics from environmental materials using FD-FLIM [24, 25]. However, these studies analyzed plastics without biological contamination, which implies an intensive cleaning process before applying findings to the examination of real-case environmental samples. In this study, plastic samples of ABS and PET were coated with peptides, bacteria, and filamentous fungus in order to test the influence of organic layers on plastic analysis using FD-FLIM. We found that ABS and PET could be distinguished based on their characteristic fluorescence lifetimes, even in the presence of organic contamination. These findings extend the research of Wohlschläger, emphasizing the robustness of FD-FLIM and the ability to discern subtle differences in fluorescence lifetimes, allowing for the reliable differentiation of plastic. This study, therefore, highlights the potential of FD-FLIM as a valuable tool in environmental monitoring, addressing the possibility of in situ MP analysis without a significant cleaning process. The results can also be translated to possible future developments of FD-FLIM, such as confocal laser scanning microscopes, handheld devices or larger scaled setups. However, some limitations are worth noting. The results of the averaged fluorescence spectrum intensity showed high standard deviations. Possible reasons for this could be the exposure time of 2 s as well as defocused areas caused by the round shape of the granules. Although the samples with defined organic layers could be distinguished, the influence of longer-term incubation, aging, and undefined multilayer environment coating on the fluorescence lifetime of plastics has not been addressed. Additionally, it was not possible to establish a quantitative limit for plastic identification in relation to the layer thicknesses or the mass ratio of the plastic to the organic layer. The influence of MP degradation on FD-FLIM measurements has yet to be addressed. A possible attenuation of the fluorescence intensity of the sample due to degradation would impact the intensity-dependent modulation lifetime and result in an increase in measurement uncertainty. However, in this research, no significant changes in the measurement results were observed due to optical irradiation. Future research should include extending the incubation periods and (multi-)coating with different phytoplankton species as well as further contamination (e.g., soil, sediment particles) to address quantitative limits of plastic identification using FD-FLIM. The experiment should also be repeated with different types of polymers and additives as well as micrometer-sized MP to verify the results. Conducting studies under different environmental conditions (e.g., varying temperatures, humidity levels, and exposure to sunlight) to simulate real-world scenarios will further assess their influence on fluorescence signals. By addressing these aspects and comparing FD-FLIM with state-of-the-art techniques, the robustness and applicability of FD-FLIM for detecting plastics under organic layers in environmental samples could be significantly enhanced.

## Supplementary Information

Below is the link to the electronic supplementary material.

Supplementary file 1 (DOCX 5.76 MB)

## Data Availability

The authors declare that the data supporting the findings of this study are available within the paper and its Supplementary Information files. Should any raw data files be needed in another format, they are available from the corresponding author upon reasonable request.
