# Supplementary material for: Effects of defined organic layers on the fluorescence lifetime of plastic materials
Source: Anal Bioanal Chem. 2025 May 9;417(16):3651–63. doi: 10.1007/s00216-025-05888-y (PMC12206207; doi:10.1007/s00216-025-05888-y)
Supplement: Supplementary file 1 — (DOCX 5.76 MB) [file 216_2025_5888_MOESM1_ESM.docx]

## Supporting Information

Manuscript: **Effects of Defined Organic Layers on the Fluorescence Lifetime of Plastic Materials**

Nina Leiter^1^, Maximilian Wohlschläger^1^, Martin Versen^1^, Sonja D. Harter^2^, Tina Kießlich^2^, Franziska Lederer^2^, Stefanie Clauß^3^, Dietmar Schlosser^3^, Emanuel Gheorghita Armanu^4^, Christian Eberlein^4^, Hermann J. Heipieper^4^, Martin G.J. Löder^5^, Christian Laforsch^5^

^1^ Faculty of Engineering Sciences, Rosenheim Technical University of Applied Sciences, Hochschulstraße 1, 83024 Rosenheim, Germany

^2^ Biotechnology Department, Helmholtz Institute Freiberg for Resource Technology, Helmholtz-Zentrum Dresden-Rossendorf, Bautzner Landstraße 400, 01328 Dresden, Germany

^3^ Department of Applied Microbial Ecology, Helmholtz Centre for Environmental Research – UFZ, Permoserstraße 15, 04318 Leipzig, Germany

^4^ Department of Molecular Environmental Biotechnology, Helmholtz Centre for Environmental Research – UFZ, Permoserstraße 15, 04318 Leipzig, Germany

^5^ Animal Ecology I and BayCEER, University Bayreuth, Universitätsstraße 30, 95440 Bayreuth, Germany

The supporting information consists of additional information on the results of the spectral fluorescence measurements (**SI section 4.1. Fluorescence spectroscopy**), additional information on the results of the fluorescence lifetime) **SI section 4.2. FD-FLIM**), and additional screenshots of FD-FLIM measurements of peptides and protein on ABS and PET (**SI section 4.2.1. Peptides and protein**), of bacteria on ABS and PET (**SI section 4.2.2. Bacteria**) and of fungus on ABS and PET (**SI section 4.2.3. Fungal mycelium**).

**SI section 4.1. Fluorescence spectroscopy**

Table S1 Location of the maximum emission intensity (Counts, $\boldsymbol{\lambda}_{\mathbf{max}}$) and Stokes shift ($\boldsymbol{\Delta\lambda}_{\mathbf{max}}$) of the averaged fluorescence spectrum over four measurements of ABS and PET at different peptides and protein, bacteria and fungus incubation times at 445 nm excitation

|  | Peptides and Protein | | | Bacteria | | | Fungus | | |
| --- | --- | --- | --- | --- | --- | --- | --- | --- | --- |
| Sample | **Counts [a.u.]** | $\boldsymbol{\lambda}_{\mathbf{max}}$ **[nm]** | $\boldsymbol{\Delta\lambda}_{\mathbf{max}}$ **[nm]** | **Counts [a.u.]** | $\boldsymbol{\lambda}_{\mathbf{max}}$ **[nm]** | $\boldsymbol{\Delta\lambda}_{\mathbf{max}}$ **[nm]** | **Counts [a.u.]** | $\boldsymbol{\lambda}_{\mathbf{max}}$ **[nm]** | $\boldsymbol{\Delta\lambda}_{\mathbf{max}}$ **[nm]** |
| ABS t0 | 34846±5673 | 514 | 69 | 33227±4485 | 519 | 74 | 36243±2011 | 520 | 75 |
| ABS t1 | 24174±9368 | 515 | 70 | 29980±3959 | 519 | 74 | 39857±1921 | 519 | 74 |
| ABS t2 | 40253±2179 | 513 | 68 | 26541±2996 | 519 | 74 | 43016±7828 | 521 | 76 |
| ABS t3 | 38439±3341 | 512 | 67 | 28687±5557 | 519 | 74 | 46603±5200 | 520 | 75 |
| ABS t4 | 32347±7830 | 515 | 70 | 29814±2403 | 519 | 74 | 43469±7536 | 521 | 76 |
| ABS t5 | 36167±4072 | 512 | 67 | 29523±3266 | 519 | 74 | 40130±4132 | 521 | 76 |
| ABS t6 | 34752±3147 | 512 | 67 |  |  |  | 42548±2653 | 519 | 74 |
| ABS t7 | 39219±1533 | 512 | 67 |  |  |  | 396474044 | 521 | 76 |
| PET t0 | 5540±2230 | 525 | 80 | 6075±2083 | 522 | 77 | 2643±466 | 526 | 81 |
| PET t1 | 4060±1030 | 524 | 79 | 6369±3032 | 524 | 79 | 4887±3240 | 526 | 81 |
| PET t2 | 5739±2676 | 523 | 78 | 7082±3021 | 523 | 78 | 7998±1630 | 528 | 83 |
| PET t3 | 5771±5537 | 523 | 78 | 5417±1287 | 525 | 80 | 6365±1268 | 526 | 81 |
| PET t4 | 4784±1220 | 524 | 79 | 4077±649 | 522 | 77 | 5367±2173 | 527 | 82 |
| PET t5 | 4257±2222 | 525 | 80 | 5941±1843 | 523 | 78 | 5800±1960 | 526 | 81 |
| PET t6 | 4377±818 | 523 | 78 |  |  |  | 6959±3090 | 524 | 79 |
| PET t7 | 3993±1727 | 525 | 80 |  |  |  | 4692±1319 | 526 | 81 |


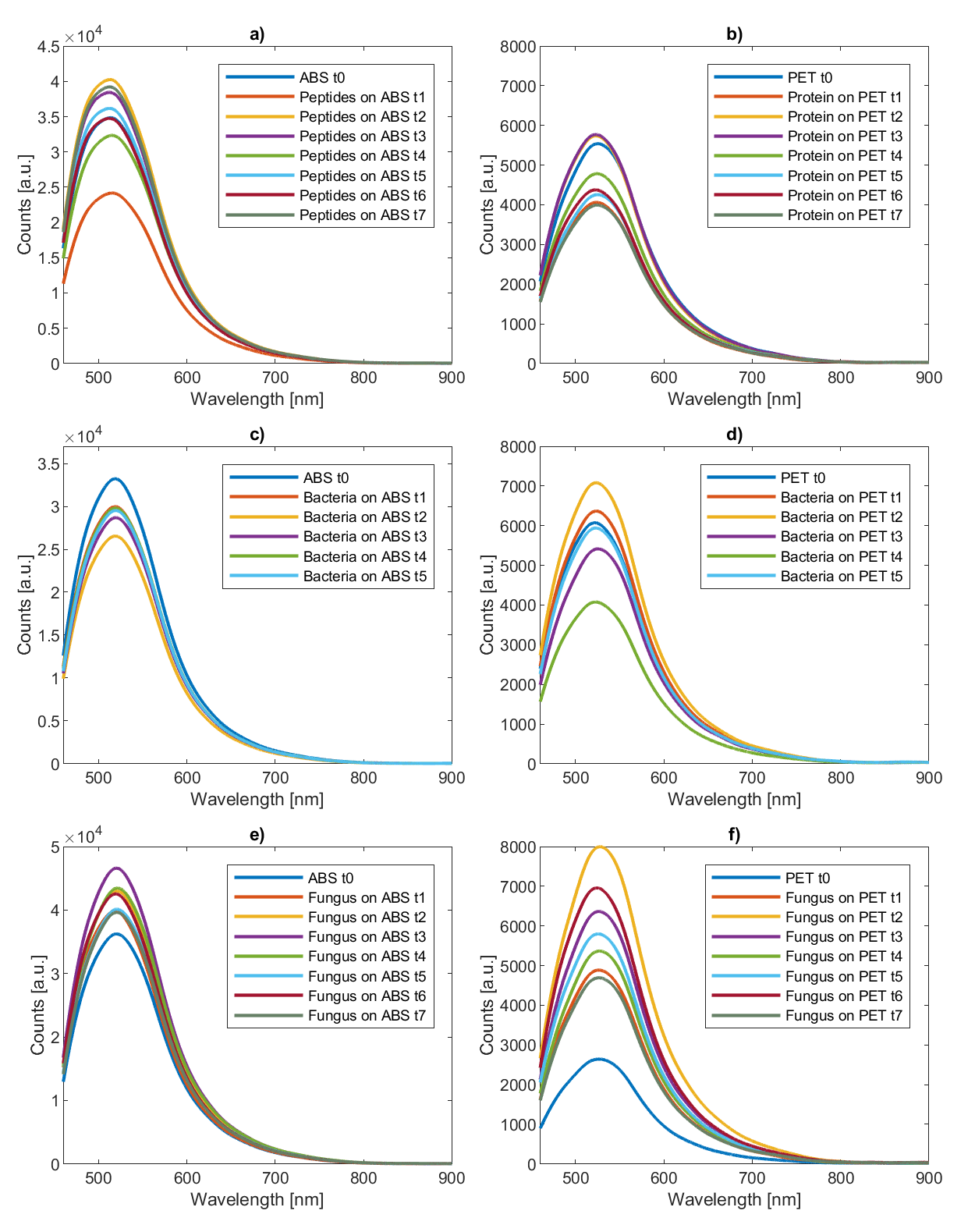


**Fig. S2** **a)** averaged fluorescence spectra of ABS extracted from peptide incubation at defined times t0 – t7; **b)** averaged fluorescence spectra of PET extracted from protein incubation at defined times t0 – t7; **c)** averaged fluorescence spectra of ABS extracted from bacteria incubation at defined times t0 – t5; **d)** averaged fluorescence spectra of PET extracted from bacteria incubation at defined times t0 – t5. **e)** averaged fluorescence spectra of ABS extracted from incubation with the fungus at defined times t0 – t7; **f)** averaged fluorescence spectra of PET extracted from incubation with the fungus at defined times t0 – t7. The maximum fluorescence intensity of the spectra does differ with respect to the incubation time. However, no pattern in intensity decrease or increase can be observed, also with respect to a wide standard deviation listed in Table S1.

**SI section 4.2. FD-FLIM**

Table S3 Calculated expectation values and standard deviations averaged from four measurements, assuming a Gaussian normal distribution from the phase-dependent ($\boldsymbol{\tau}_{\mathbf{PH1}}\boldsymbol{,}\boldsymbol{\tau}_{\mathbf{PH2}}$) and modulation-dependent ($\boldsymbol{\tau}_{\mathbf{M1}}\boldsymbol{,}\boldsymbol{\tau}_{\mathbf{M2}}$) fluorescence lifetimes with corresponding standard deviation ($\boldsymbol{1*\sigma)}$ of ABS and PET at different peptides, bacteria and fungus incubation times at 445 nm excitation

| Sample | $\boldsymbol{\tau}_{\mathbf{PH1}}$ [ns] | $\boldsymbol{\tau}_{\mathbf{PH2}}$ [ns] | $\boldsymbol{\tau}_{\mathbf{M1}}$ [ns] | $\boldsymbol{\tau}_{\mathbf{M2}}$ [ns] |
| --- | --- | --- | --- | --- |
|  | **Peptides and Protein** | | | |
| ABS t0 | 3.02±0.06 | - | 4.24±0.09 | - |
| ABS t1 | 3.03±0.06 | - | 4.17±0.13 | - |
| ABS t2 | 3.05±0.05 | - | 4.10±0.07 | - |
| ABS t3 | 3.12±0.06 | 3.01±0.04 | 4.10±0.07 | 4.30±0.09 |
| ABS t4 | 3.09±0.07 | - | 4.26±0.11 | - |
| ABS t5 | 3.12±0.06 | - | 4.31±0.09 | - |
| ABS t6 | 3.09±0.07 | - | 4.34±0.08 | 4.11±0.07 |
| ABS t7 | 3.09±0.08 | - | 4.32±0.08 | 4.11±0.07 |
| PET t0 | 2.50±0.05 | - | 3.41±0.10 | - |
| PET t1 | 2.62±0.05 | - | 3.48±0.09 | - |
| PET t2 | 2.60±0.07 | - | 3.42±0.10 | - |
| PET t3 | 2.58±.0.07 | - | 3.43±0.12 | - |
| PET t4 | 2.60±0.05 | - | 3.44±0.10 | - |
| PET t5 | 2.61±0.08 | - | 3.47±0.13 | - |
| PET t6 | 2.60±0.05 | - | 3.44±0.10 | - |
| PET t7 | 2.60±0.06 | - | 3.45±0.11 | - |
|  | **Bacteria** | | | |
| ABS t0 | 2.95±0.04 | - | 4.15±0.06 | - |
| ABS t1 | 2.96±0.05 | - | 4.17±0.08 | - |
| ABS t2 | 2.94±0.05 | - | 4.15±0.08 | - |
| ABS t3 | 2.94±0.04 | - | 4.15±0.07 | - |
| ABS t4 | 2.92±0.04 | - | 4.15±0.08 | - |
| ABS t5 | 2.91±0.04 | - | 4.12±0.07 | - |
| PET t0 | 2.56±0.05 | - | 3.52±0.10 | - |
| PET t1 | 2.63±0.06 | - | 3.55±0.10 | - |
| PET t2 | 2.64±0.05 | - | 3.57±0.11 | - |
| PET t3 | 2.66±0.05 | - | 3.60±0.10 | - |
| PET t4 | 2.66±0.05 | - | 3.60±0.10 | - |
| PET t5 | 2.64±0.05 | - | 3.57±0.10 | - |
|  | **Fungus** | | | |
| ABS t0 | 3.00±0.05 | - | 4.14±0.08 | - |
| ABS t1 | 2.96±0.06 | - | 4.12±0.09 | - |
| ABS t2 | 2.96±0.05 | - | 4.13±0.08 | 3.91±0.22 |
| ABS t3 | 2.97±0.04 | 2.85±0.05 | 4.08±0.08 | - |
| ABS t4 | 2.97±0.05 | 2.59±0.05 | 4.13±0.08 | 3.83±0.07 |
| ABS t5 | 2.93±0.04 | 2.78±0.11 | 4.05±0.11 | - |
| ABS t6 | 2.98±0.05 | 2.79±0.07 | 4.08±0.13 | - |
| ABS t7 | 2.95±0.05 | 2.84±0.07 | 4.10±0.08 | - |
| PET t0 | 2.60±0.06 | - | 3.52±0.10 | - |
| PET t1 | 2.50±.0.7 | 2.26±0.10 | 3.42±0.20 | - |
| PET t2 | 2.57±0.07 | 2.25±0.09 | 3.36±0.18 | - |
| PET t3 | 2.54±.011 | 2.20±0.05 | 3.43±0.16 | - |
| PET t4 | 2.48±0.14 | - | 3.51±0.16 | - |
| PET t5 | 2.59±0.08 | 2.03±0.08 | 3.49±0.14 | - |
| PET t6 | 2.61±0.07 | - | 3.46±0.12 | - |
| PET t7 | 2.61±0.09 | 2.28±0.14 | 3.43±0.19 | - |

In Fig. S4-S9 exemplary FD-FLIM measurements using pco.flim camera are shown by screenshots of the NIS-elements software. The area of measurement of the pco.flim camera contains 1004x1008 pixels. Considering the field of view of (0.28 x 0.28) mm^2^ at a magnification of x20 and the detector pixel size of the pco.flim camera of 5.6 µm, a pixel resolution of 0.28 µm is given. The fluorescence intensity (intensity) is shown in greyscale, while phase-shift (phase), modulation index (modulation), phase-dependent fluorescence lifetime (phase lifetime), and modulation-dependent fluorescence lifetime (modulation lifetime) are displayed as false color images. The fluorescence intensity images show defocused areas due to the round shape of the samples. Possible intensity degradation does not impact the phase-dependent fluorescence lifetime. However, since the modulation-dependent fluorescence lifetime is described by the amplitude of excitation the amplitude of the fluorescence emission , the average value of excitation intensity and the average value of the fluorescence emission intensity, a possible intensity degradation results in an increase of the modulation-dependent fluorescence lifetime uncertainty.


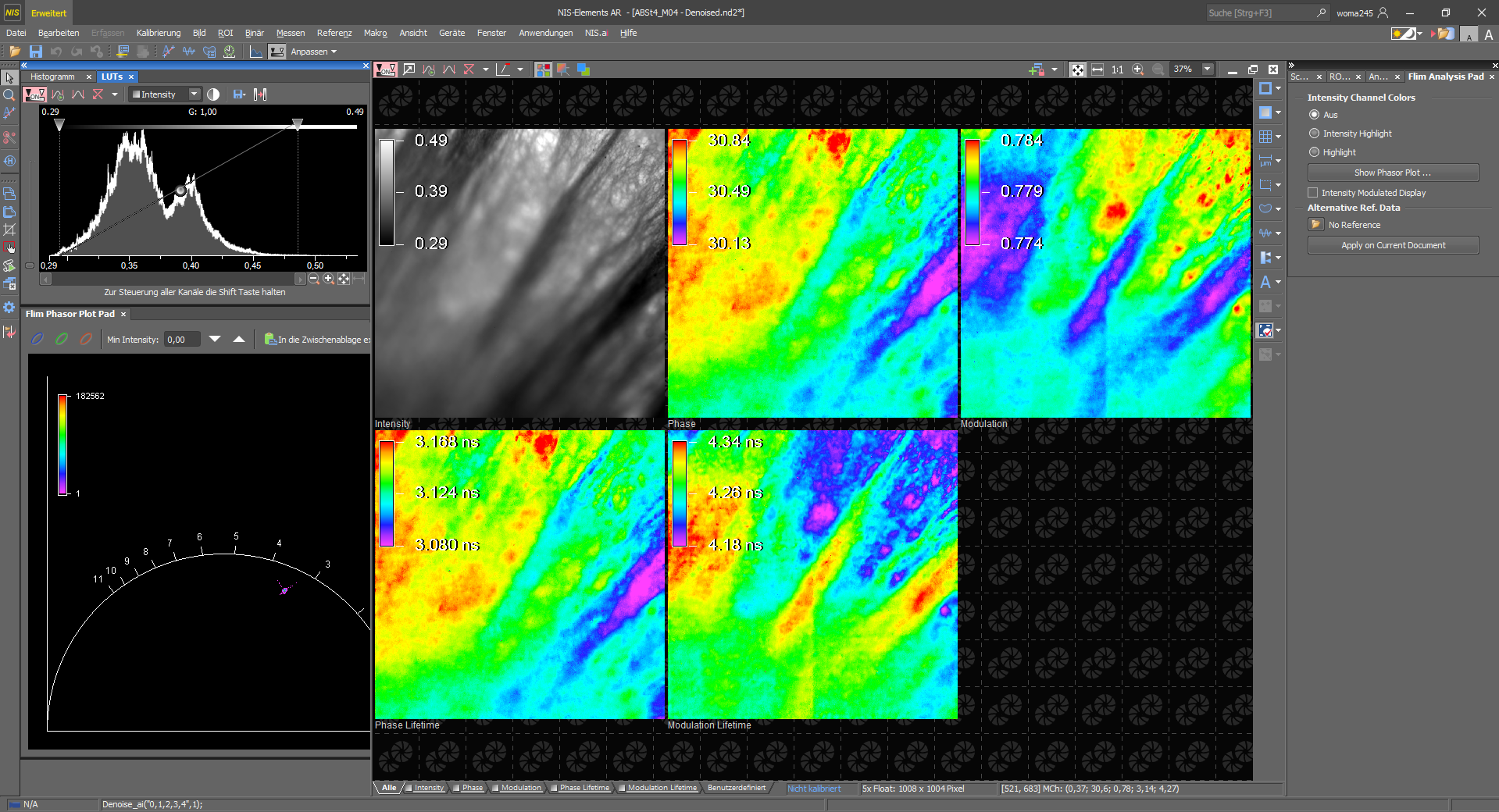
**SI section 4.2.1. Peptides and protein**

**50 µm**

**Fig. S4** Screenshot of an exemplary FD-FLIM measurement of ABS with peptides at t4 resulting in a stack of images containing fluorescence intensity (intensity), phase-shift (phase), modulation index (modulation), phase-dependent fluorescence lifetime (phase lifetime), and modulation-dependent fluorescence lifetime (modulation lifetime)


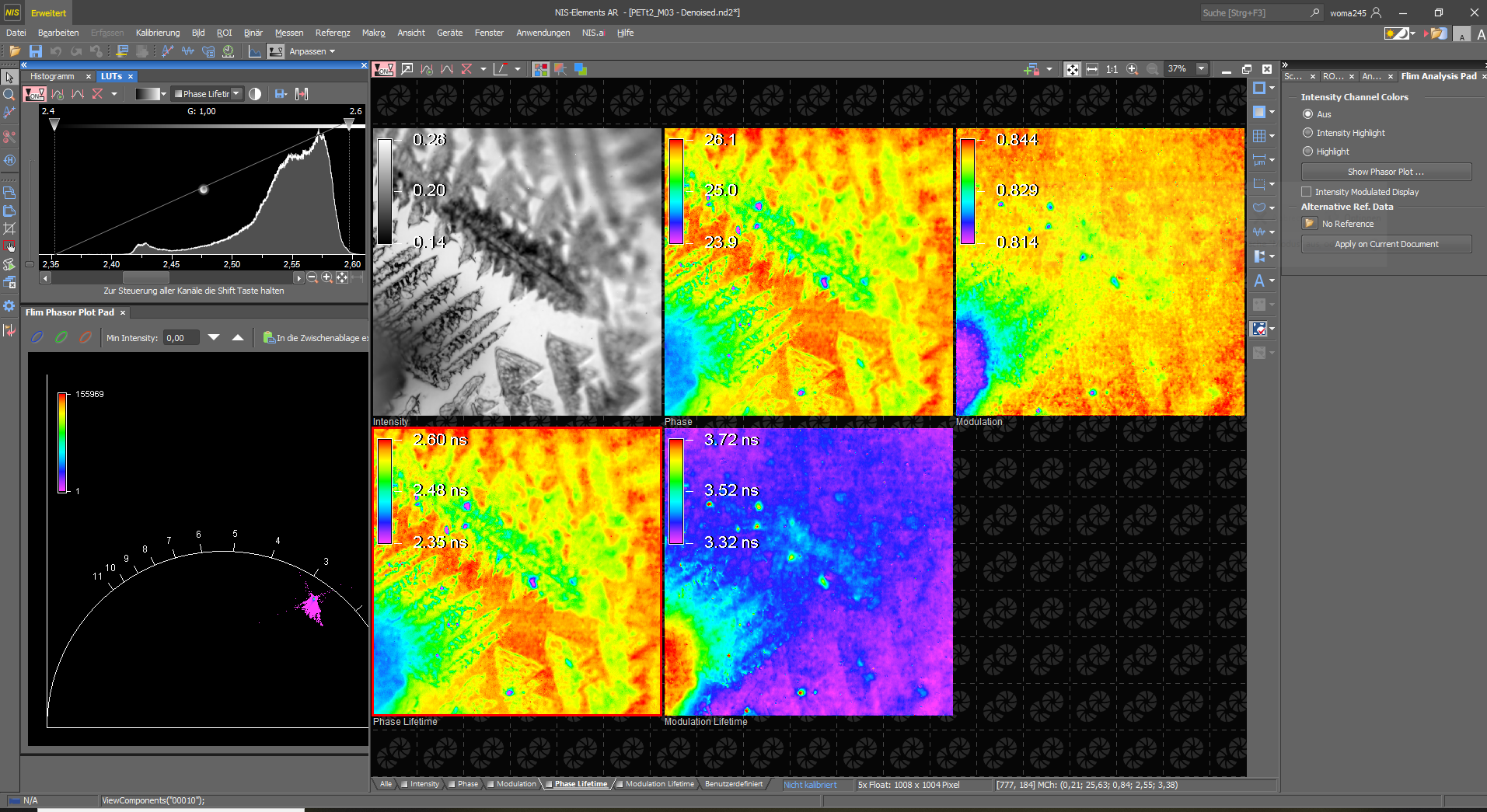


**50 µm**

**Fig. S5** Screenshot of an exemplary FD-FLIM measurement of PET with BSA at t2 resulting in a stack of images containing fluorescence intensity (intensity), phase-shift (phase), modulation index (modulation), phase-dependent fluorescence lifetime (phase lifetime), and modulation-dependent fluorescence lifetime (modulation lifetime)


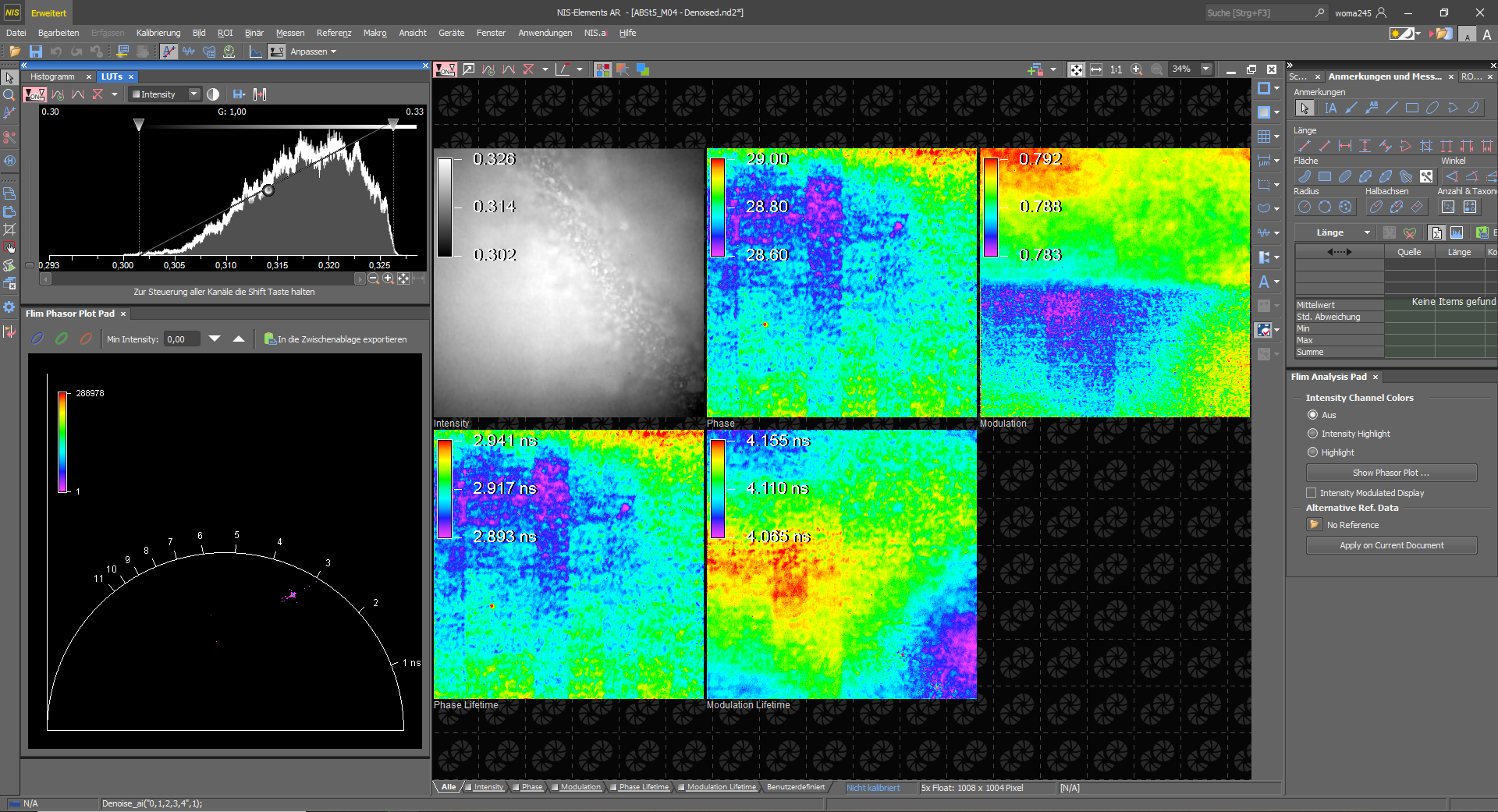
**SI section 4.2.2. Bacteria**

**50 µm**

**Fig. S6** Screenshot of an exemplary FD-FLIM measurement of ABS with bacteria at t5 resulting in a stack of images containing fluorescence intensity (intensity), phase-shift (phase), modulation index (modulation), phase-dependent fluorescence lifetime (phase lifetime), and modulation-dependent fluorescence lifetime (modulation lifetime); sharp edges of rectangular, vertical regions result from sensor artifacts of the third counting digit caused by a structurally weak surface in combination with closely spaced fluorescence lifetimes

**
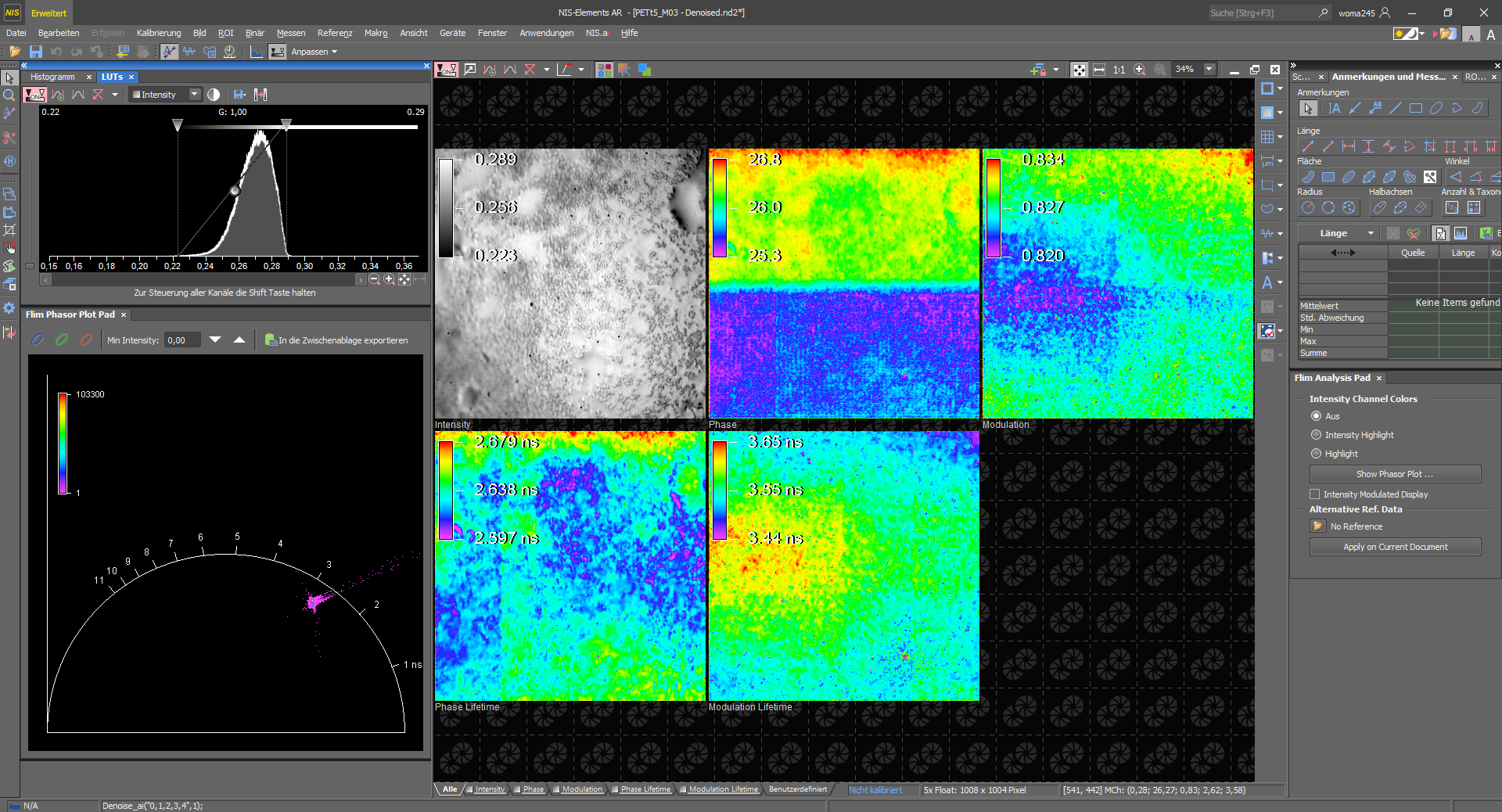
**

**50 µm**

**Fig. S7** Screenshot of an exemplary FD-FLIM measurement of PET with bacteria at t5 resulting in a stack of images containing fluorescence intensity (intensity), phase-shift (phase), modulation index (modulation), phase-dependent fluorescence lifetime (phase lifetime), and modulation-dependent fluorescence lifetime (modulation lifetime) of PET with bacteria at t5; sharp edges of rectangular regions result from sensor artifacts of the third counting digit caused by a structurally weak surface in combination with closely spaced fluorescence lifetimes


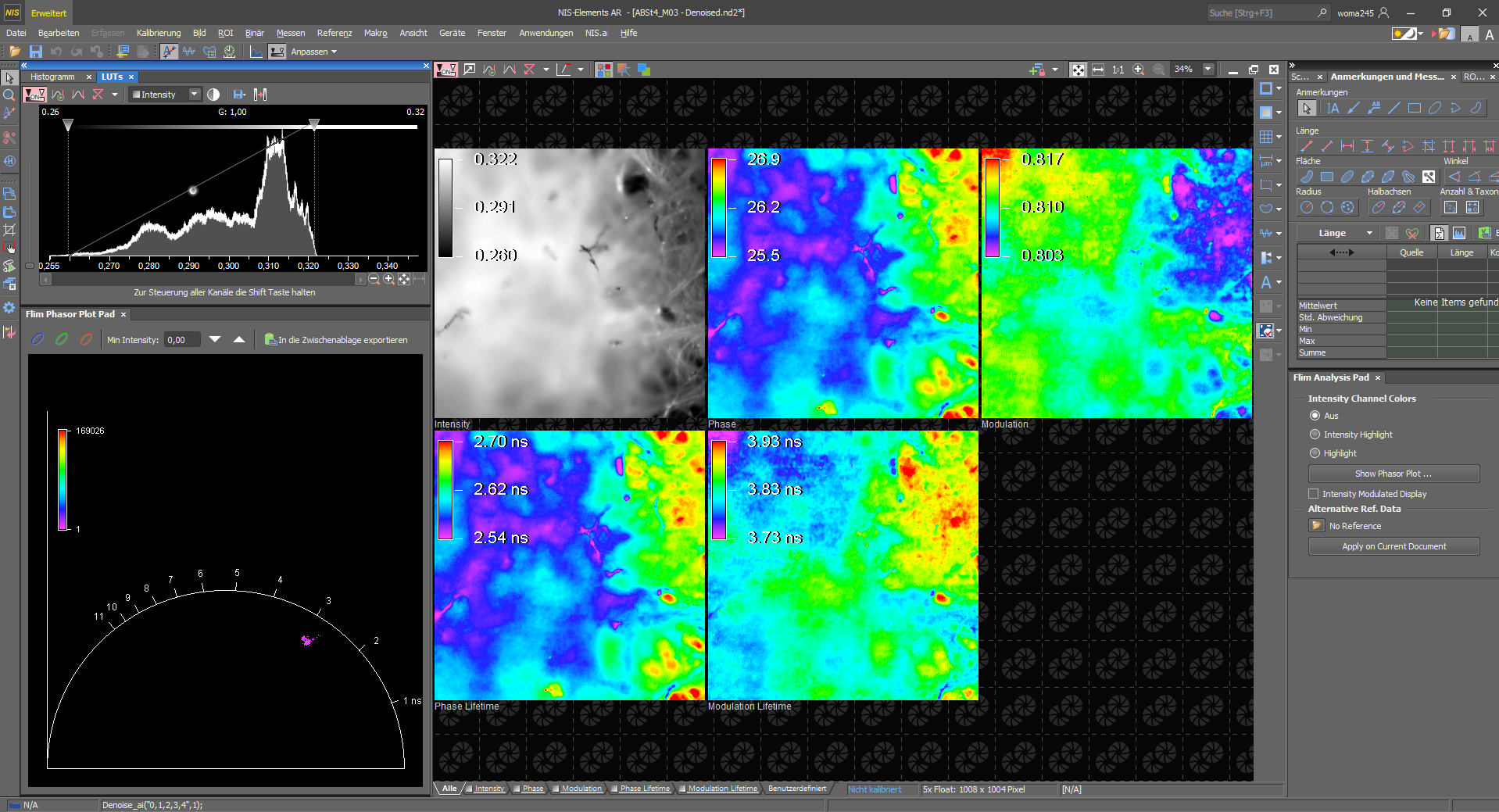
**SI section 4.2.3. Fungal mycelium**

**50 µm**

**Fig. S8** Screenshot of an exemplary FD-FLIM measurement of ABS with the fungus at t4 resulting in a stack of images containing fluorescence intensity (intensity), phase-shift (phase), modulation index (modulation), phase-dependent fluorescence lifetime (phase lifetime), and modulation-dependent fluorescence lifetime (modulation lifetime)


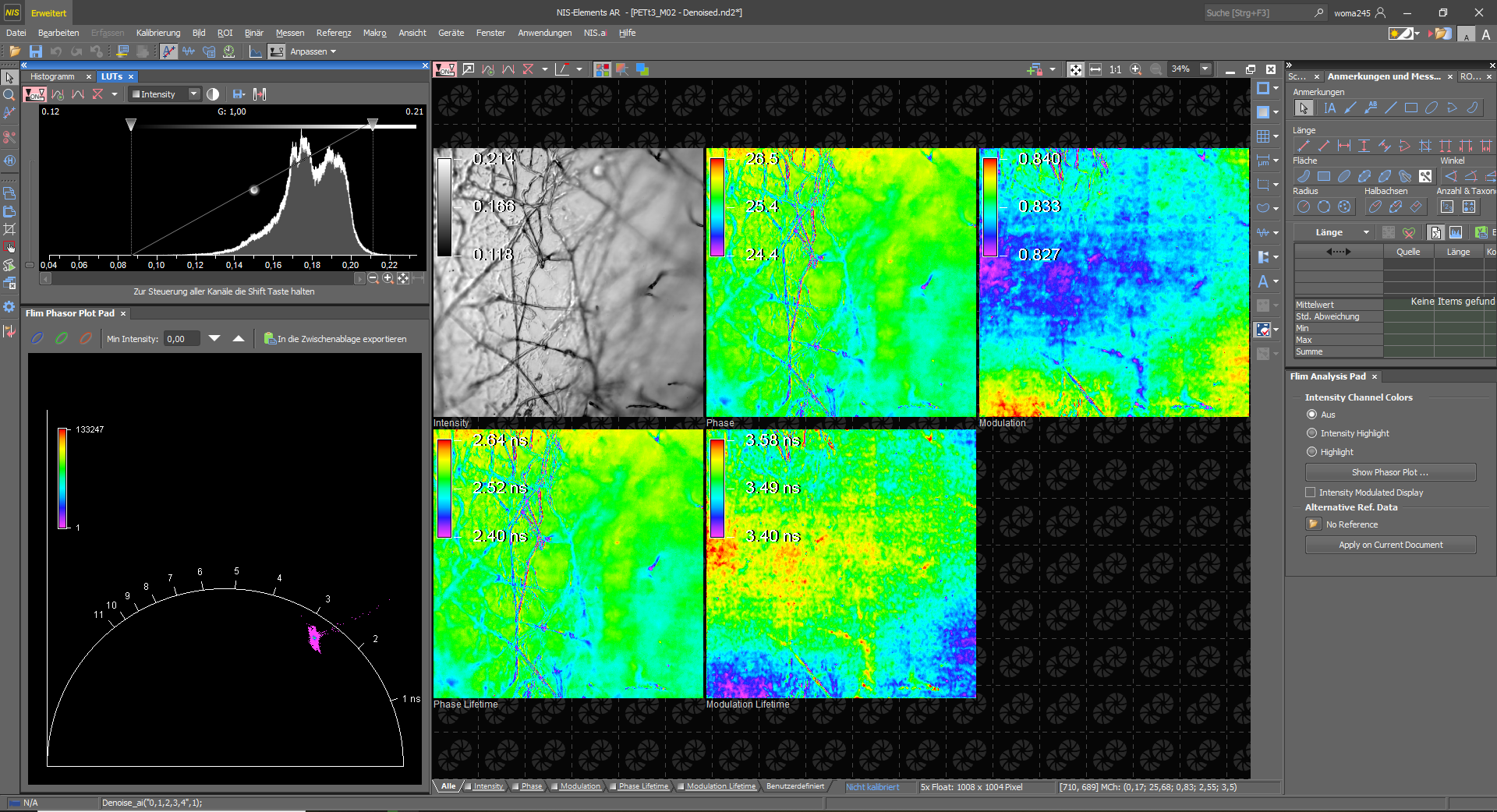


**50 µm**

**Fig. S9** Screenshot of an exemplary FD-FLIM measurement of PET with the fungus at t3 resulting in a stack of images containing fluorescence intensity (intensity), phase-shift (phase), modulation index (modulation), phase-dependent fluorescence lifetime (phase lifetime), and modulation-dependent fluorescence lifetime (modulation lifetime)
